# *In Vitro* and *in* *Vivo* Antitumor Effect of Trachylobane-360, a Diterpene from *Xylopia langsdorffiana*

**DOI:** 10.3390/molecules17089573

**Published:** 2012-08-10

**Authors:** João Carlos Lima Rodrigues Pita, Aline Lira Xavier, Tatyanna Kelvia Gomes de Sousa, Vivianne Mendes Mangueira, Josean Fechine Tavares, Robson José de Oliveira Júnior, Robson Cavalcante Veras, Hilzeth de Luna Freire Pessoa, Marcelo Sobral da Silva, Sandra Morelli, Veridiana de Melo Rodrigues Ávila, Teresinha Gonçalves da Silva, Margareth de Fátima Formiga Melo Diniz, Marianna Vieira Sobral Castello-Branco

**Affiliations:** 1 Laboratory of Pharmaceutical Technology, Federal University of Paraíba, P.O. Box 5009, João Pessoa 58051-970, PB, Brazil; Email: joaocpita@yahoo.com.br (J.C.L.R.P.); alinelx_farma@yahoo.com.br (A.L.X.); tatyannakelvia@hotmail.com (T.K.G.S.); viviannemendes_cz@hotmail.com (V.M.M.); josean@ltf.ufpb.br (J.F.T.); robveras@msn.com (R.C.V.); hilzeth@gmail.com (H.L.F.P.); marcelosobral@ltf.ufpb.br (M.S.S.); margareth@ccs.ufpb.br (M.F.F.M.D.); 2 Laboratory of Cytogenetic, Institute of Genetics and Biochemistry, Federal University of Uberlandia, P.O. Box 593, Uberlândia 38400-902, MG, Brazil; Email: robson_junr@yahoo.com.br (R.J.O.J.); morelli@ufu.br (S.M.); veridiana@ingeb.ufu.br (V.M.R.A.); 3 Departament of Antibiotics, Federal University of Pernambuco, Recife 50670-901, PE, Brazil; Email: teresinha100@gmail.com

**Keywords:** trachylobane-360, *Xylopia langsdorffiana*, antitumor activity, toxicity, hemolytic activity

## Abstract

Trachylobane-360 (*ent*-7α-acetoxytrachyloban-18-oic acid) was isolated from *Xylopia langsdorffiana*. Studies have shown that it has weak cytotoxic activity against tumor and non-tumor cells. This study investigated the *in vitro* and *in vivo* antitumor effects of trachylobane-360, as well as its cytotoxicity in mouse erythrocytes. In order to evaluate the *in vivo* toxicological aspects related to trachylobane-360 administration, hematological, biochemical and histopathological analyses of the treated animals were performed. The compound exhibited a concentration-dependent effect in inducing hemolysis with HC_50_ of 273.6 µM, and a moderate *in vitro* concentration-dependent inhibitory effect on the proliferation of sarcoma 180 cells with IC_50_ values of 150.8 µM and 150.4 µM, evaluated by the trypan blue exclusion test and MTT reduction assay, respectively. The *in vivo* inhibition rates of sarcoma 180 tumor development were 45.60, 71.99 and 80.06% at doses of 12.5 and 25 mg/kg of trachylobane-360 and 25 mg/kg of 5-FU, respectively. Biochemical parameters were not altered. Leukopenia was observed after 5-FU treatment, but this effect was not seen with trachylobane-360 treatment. The histopathological analysis of liver and kidney showed that both organs were mildly affected by trachylobane-360 treatment. Trachylobane-360 showed no immunosuppressive effect. In conclusion, these data reinforce the anticancer potential of this natural diterpene.

## 1. Introduction

Cancer chemotherapeutic strategies should be developed to provide higher tumor response and lower toxicity. However, commonly used cytotoxic chemotherapy is largely associated with highly nonspecific cytotoxicity, narrow therapeutic indices, and undesirable side effects [1].

The study of natural products has been the single most successful strategy for the discovery of new medicines used in anticancer therapy, such as vindesine, vinorelbine, etoposide, docetaxel and topotecan [2]. In addition, several other natural products are being tested for antitumor activity [3,4].

**Figure 1:**
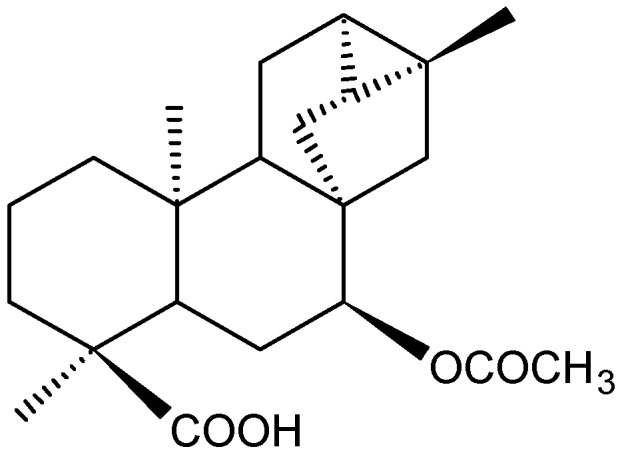
Trachylobane-360 isolated from *Xylopia langsdorffiana*.

*Xylopia langsdorffiana* St-Hil. & Tul. (Annonaceae) is a 5–7 m high tree popularly known in Northeast Brazil as “pimenteira da terra” [5]. Trachylobane-360 (Figure 1) was isolated from *Xylopia langsdorffiana* stems and characterized as a new diterpene, *ent*-7α-acetoxytrachyloban-18-oic acid (molar mass = 359.47) [6]. Trachylobane diterpenes are secondary metabolites, quite rare in Nature, and their bioactivities are poorly understood. However, other trachylobane-type diterpenes isolated from different species have demonstrated antitumor [7,8,9], vasorelaxant [10], antimicrobial [9] and antiosteoclastogenic activities [11]. In addition, Block *et al.* [12] showed that a trachylobane diterpene was able to induce apoptosis in human promyelocytic leukemia cells via caspase-3 activation. We have previously reported that trachylobane-360 inhibits cell growth and induces differentiation in human leukemia cell lines (HL60, U937 and K562) [13]. Moreover, trachylobane-360 shows weak cytotoxicity against V79 cells and rat hepatocytes [6]. Although some studies show that this class of substances has antitumor potential *in vitro* in different cell lines of [9,12,13,14], there are no prior studies in the literature of antitumor activity *in vivo* with any of them.

The aim of this study was to investigate, in experimental models, the *in vitro* and *in vivo* antitumor effects of trachylobane-360 isolated from the stems of *X. langsdorffiana*, as well as its cytotoxic effects on mouse erythrocytes. In order to evaluate the *in vivo* toxicological aspects related to trachylobane-360 administration, hematological, biochemical and histopathological analyses of the treated animals were performed.

## 2. Results and Discussion

### 2.1. Hemolysis Assay

**Figure 2:**
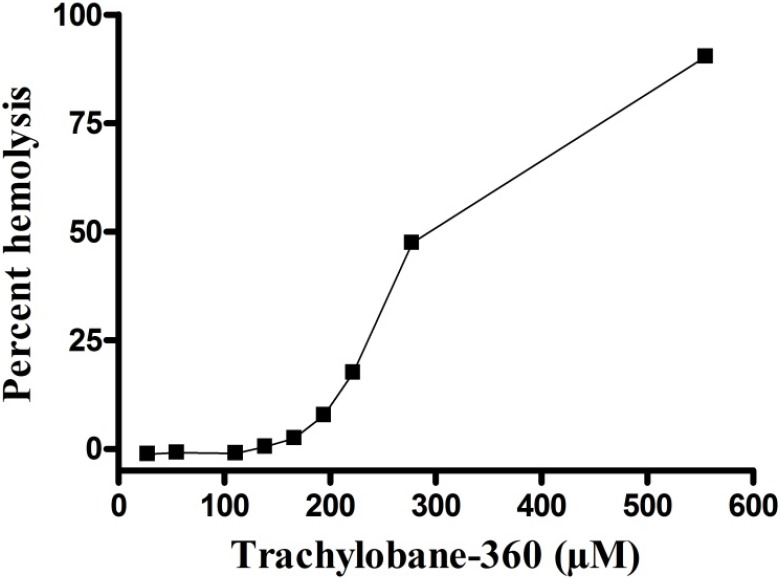
Hemolytic action of trachylobane-360 of Xylopia langsdorffiana. Values are means ± SEM from three independent experiments.

The compound exhibited a significant concentration-dependent hemolytic effect (Figure 2). The HC_50_ value was 273.6 (273.2–274.0) µM.

**Figure 3:**
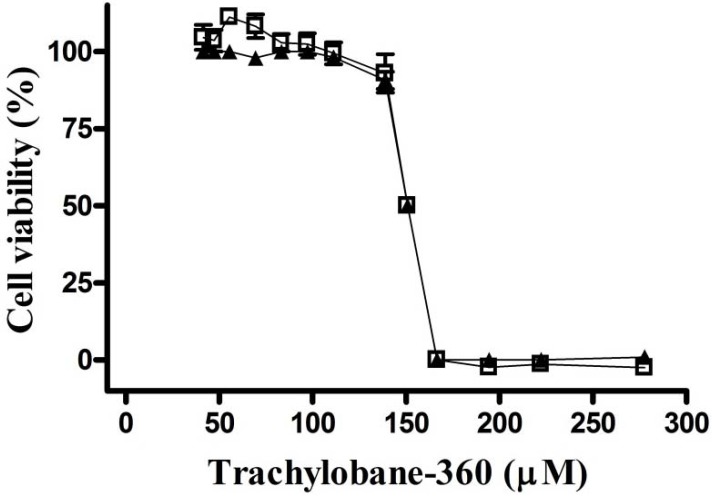
Cell viability after treatment with trachylobane-360. Concentration-response curve with two cytotoxicity assays: (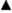) trypan blue exclusion test and (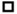) MTT reduction. Each point represents the mean ± SEM of three experiments in four replicates, with 95% confidence intervals.

The mechanical stability of erythrocyte membrane is a good indicator of *in vitro* damage in cytotoxicity assay, since drugs can alter this delicate structure [15]. Red cells also provide a preliminary model to study protective effects and toxic of substances or conditions associated with oxidative stress, where it is a possible indicator of such damage to cells [16,17,18,19]. The results of the toxicity of trachylobane-360 against sarcoma 180 cells, suggest that oxidative stress may have been involved, at least in part, because at a concentration of 166.67 µM, which produced 100% death of tumor cells (Figure 3) and 2.42% hemolysis (Figure 2). Additional experiments are needed to quantify the involvement of oxidative stress in the cytotoxicity mechanism of trachylobane-360.

### 2.2. Antitumor Activity of Trachylobane-360

#### 2.2.1. Effect of Trachylobane-360 on Tumor Cell in Culture

The *in vitro* effect of trachylobane-360 against sarcoma 180 tumor cell line was determined. The compound exhibited a moderate concentration-dependent inhibitory effect on the proliferation of sarcoma 180 cells. The IC_50_ values were 150.8 (150.5–151.1) µM and 150.4 (149.8–151.1) µM, evaluated by the trypan blue exclusion test and MTT reduction assay, respectively (Figure 3).

The *in vitro* antitumor activity was evaluated by cytotoxicity assays using the trypan blue exclusion and MTT reduction assays. Recent studies have shown that some substances are somehow able to interact with MTT and thus inhibit MTT reduction without affecting cell viability, or induce the reduction of MTT in the absence of living cells. Therefore, the use of other cytotoxicity assays, in addition to the MTT reduction, is recommended to evaluate the effect of chemicals on cell growth *in vitro*, including the trypan blue exclusion assay [20,21].

The present data demonstrate that trachylobane-360 inhibited the proliferation of sarcoma 180 cells in both cytotoxicity assays. The two methods of evaluation of cytotoxicity provided similar IC_50_ values, with their curves almost overlapping point to point. Thus, it appears that there was no interference, as mentioned above, with the MTT reduction method (Figure 3). The cytotoxicity of trachylobane-360 was previously evaluated in V79 cells (Chinese hamster lung fibroblasts) and rat hepatocytes by the MTT reduction assay, giving IC_50_ values of 224 and 231 µM, respectively [6]. Comparing these results to the IC_50_ value obtained for sarcoma 180, trachylobane-360 appears to be more cytotoxic to tumor cells than non neoplastic normal cells. Corroborating these results, Castello-Branco *et al.* [13] showed that trachylobane-360 is more cytotoxic in human leukemia cells (HL60, U937 and K562) than in hepatocytes and V79. Since cytotoxic substances are not selectively lethal to tumor cells and substantially affect normal cellular structures, there is a constant search for new chemotherapeutic drugs with higher antitumor activity and less toxic effects to normal cells.

#### 2.2.2. Effect of Trachylobane-360 on Tumor Growth *in vivo*

**Figure 4:**
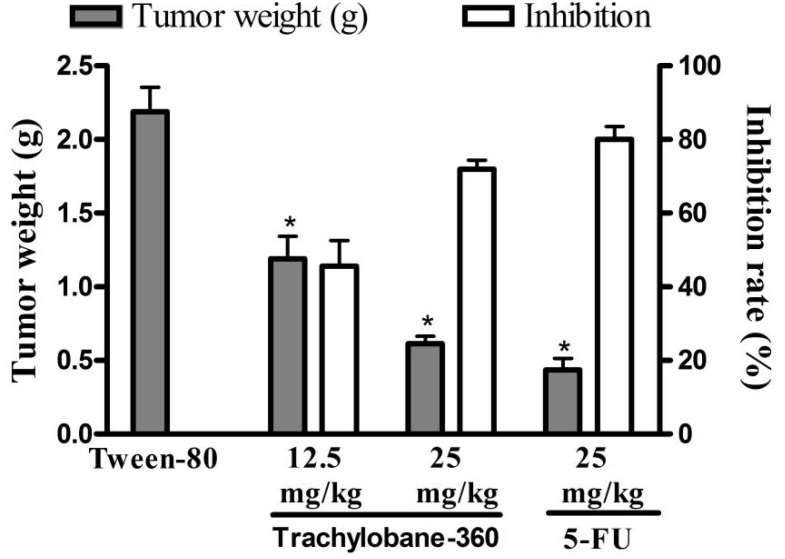
Effect of trachylobane-360 isolated from *Xylopia langsdorffiana* and 5-FU on mice transplanted with sarcoma 180 tumor. The graph shows the tumor weight (g) and the tumor growth inhibition rates. Data are presented as means ± SEM of six animals. * *p* < 0.05 compared to control-S180 (5% Tween-80) by ANOVA followed by Tukey’s test.

The effects of trachylobane-360 on mice inoculated with sarcoma 180 tumor are presented in Figure 4. There was a significant reduction in tumor weight in trachylobane-360-treated animals at both doses (*p* < 0.05). On day 8, the average tumor weight of the control mice inoculated with sarcoma 180 was 2.19 ± 0.17 g. In the presence of trachylobane-360, the sarcoma 180 weight was reduced to 1.19 ± 0.15 and 0.61 ± 0.05 g at doses of 12.5 and 25 mg/kg, respectively. These weight reductions gave inhibition rates of 45.60% and 71.99%. At 25 mg/kg, 5-FU reduced tumor weight by 80.06% within the same period.

Sarcoma 180 is a mouse-derived tumor and one of the most frequently used cell lines in antitumor related research *in vivo*, including natural products research [22,23,24]. Trachylobane-360 inhibited tumor growth *in vivo* in a dose-dependent manner, reducing the weight of the tumor, with no significant difference between the inhibition rate observed with the higher dose (25 mg/kg) of diterpene and that observed with the chemotherapeutic agent 5-FU (Figure 4). Since 1957, 5-FU has played an important role in the treatment of colon cancer and is used by patients with breast cancer and head and neck cancer [25].

**Figure 5:**
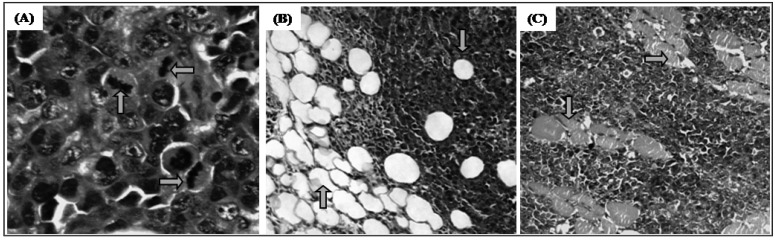
Histopathology of tumors of different experimental groups: (**A**) Asymmetric mitoses; (**B**) Invasion of adipose tissue; (**C**) Invasion of skeletal muscle tissue. Histological sections stained with hematoxylin-eosin (×400 for **A**, ×100 for **B** and **C**).

Histopathological analysis of the tumors extirpated from mice showed malignant neoplastic growth of high degree, with a solid consistency, coagulative necrosis areas and asymmetric mitosis, where these feature varied between groups. It was also observed that this tumor naturally invades adipose tissue and skeletal muscle without producing metastases in the organs studied (liver, kidneys and heart), showing groups of large, round and polygonal cells, with pleomorphic shapes and hyperchromatic nuclei (Figure 5). In the tumors extirpated from control mice were counted about eight mitoses and limited areas of coagulative necrosis were observed. Tumors of animals treated with 5-FU (25 mg/kg) showed four to five mitoses with large areas of coagulative necrosis associated with polymorphonuclear exudate. However, on analysis of tumors from animals treated with 12.5 and 25 mg/kg trachylobane-360 three to four and two to three asymmetric mitosis were observed, respectively, with extensive areas of coagulative necrosis, where there were islands of tumor cells.

##### 2.2.2.1. Toxicological Analyses

###### 2.2.2.1.1. Effects of Trachylobane-360 on Organ and Body Weight

After sacrificing the animals, the organs were removed and weighed. No significant changes in liver or kidney weights were seen between control-S180 and healthy animals (Table 1). However, spleen and thymus weights were significantly increased in animals inoculated with sarcoma 180 cells, when compared to the healthy group. After 5-FU treatment, the spleen, thymus and liver weights were significantly reduced when compared to the control-S180 group. When animals received trachylobane-360, the spleen, thymus and liver weights were similar to those of control animals and differed from those treated with 5-FU. No significant changes in body weight gain were seen among the groups treated with trachylobane-360, the control-S180 group and healthy mice. Meanwhile, animals treated with 5-FU showed weight reduction when compared to control animals.

**Table 1:** Effects of trachylobane-360 isolated from *Xylopia langsdorffiana* on organ weights. Mice were inoculated with sarcoma 180 cells (25 × 10^6^ cells/mL, s.c.). The animals were treated for seven consecutive days, starting one day after tumor implantation.

Drug	Dose (mg/kg)	Thymus (mg/g body weight)	Spleen (mg/g body weight)	Liver (mg/g body weight)	Kidney (mg/g body weight)	Initial weight (g)	Final weight (g)
Healthy mice							
(5% Tween-80)	-	3.09 ± 0.30	5.21 ± 0.24	56.33 ± 0.49	12.23 ± 0.74	32.07 ± 0.79	32.38 ± 0.62
Control-S180							
(5% Tween-80)	-	4.36 ± 0.50 ^c^	6.85 ± 0.50 ^c^	63.30 ± 1.17	10.62 ± 0.13	31.43 ± 0.99	34.54 ± 0.90
5-FU	25	2.13 ± 0.11 ^a^	5.24 ± 0.15 ^a^	57.52 ± 0.92 ^a^	10.67 ± 0.59	30.27 ± 0.49	30.35 ± 0.73 ^a^
Trachylobane-360	12.5	3.80 ± 0.31 ^b^	7.62 ± 0.37 ^b,c^	63.04 ± 1.60	11.39 ± 0.28	32.10 ± 0.68	33.77 ± 0.70
Trachylobane-360	25	3.41 ± 0.16 ^b^	7.59 ± 0.36 ^b,c^	58.75 ± 1.07	10.96 ± 0.56	29.73 ± 0.50	31.37 ± 0.53 ^a^

Data are presented as means ± SEM for six animals. ^a^ *p* < 0.05, compared to control-S180, by ANOVA followed by Tukey’s test; ^b^ *p* < 0.05, compared to mice transplanted with S180 tumor and treated with 5-FU, by ANOVA followed by Tukey’s test; ^c^ *p* < 0.05, compared to healthy mice, by ANOVA followed by Tukey’s test.

The immune system is a network of cells and organs that work together to defend the body against attacks by intruders. When the spleen suffers damage or is removed, the individual is more susceptible to infections [26]. The thymus provides resistance against the development of tumors in animals [27]. The results showed that the weight of the spleen and thymus significantly increased in animals inoculated with sarcoma 180 when compared to healthy non-transplanted animals (Table 1), corroborating data reported by Lins *et al.* [28]. Eichhorst *et al.* [29] showed that 5-FU causes apoptosis in different cell types including hepatocytes, thymocytes and splenocytes, which is partially mediated by the CD95 system due to an up-regulation of CD95 and CD95L, causing liver, thymus and spleen involution, which agrees with our results. However, trachylobane-360 did not alter these parameters. Therefore, the data show that trachylobane-360 has no immunostimulant effect, nor does it produce immunosuppression, which is a major side effect of most chemotherapy drugs currently used in clinical practice. In assessing the effects on the animal’s body weight, it was found that there was a significant decrease in weight only in the group treated with 5-FU, which corroborates findings in the literature [30].

###### 2.2.2.1.2. Effect of Trachylobane-360 on Biochemical Parameters

No significant changes in renal (urea and creatinine levels) or liver parameters (enzymatic activity of transaminases AST and ALT) were seen in animals treated with trachylobane-360 or with 5-FU in Sarcoma 180 tumor transplanted mice (Table 2).

**Table 2:** Effects of trachylobane-360 isolated from *Xylopia langsdorffiana* on biochemical parameters determined in peripheral blood. Mice were inoculated with sarcoma 180 cells (25 × 10^6^ cells/mL, s.c.). The animals were treated for seven consecutive days, starting one day after tumor implantation.

Drug	Dose (mg/kg)	AST (UI/L)	ALT (UI/L)	Urea (mg/dL)	Creatinine (mg/dL)
Healthy mice					
(5% Tween-80)	-	217.6 ± 26.2	122.6 ± 19.1	41.2 ± 2.2	0.28 ± 0,02
Control-S180					
(5% Tween-80)	-	287.8 ± 20.2	71.8 ± 7.3	39.8 ± 4.4	0.28 ± 0.02
5-FU	25	219.7 ± 18.4	63.8 ± 9.7	48.0 ± 3.5	0.32 ± 0.02
Trachylobane-360	12.5	275.0 ± 15.5	76.0 ± 10.4	48.8 ± 5.8	0.36 ± 0.06
Trachylobane-360	25	298.3 ± 13.5	109.0 ± 10.1	51.2 ± 4.7	0.40 ± 0.04

Data are presented as means ± SEM of six animals.

The liver is the organ for detoxification in mammals, and the kidneys is the most important excretory organ, therefore both are susceptible to damage by chemotherapy drugs. Examples that can be cited are mithramycin-induced liver dysfunction and renal toxicity caused by docetaxel [31]. According to the results, no significant change in levels of urea, creatinine, AST and ALT were seen, and there was no evidence of renal or hepatic damage (Table 2).

###### 2.2.2.1.3. Effect of Trachylobane-360 on Hematological Parameters

No significant changes in the parameters mean corpuscular volume (MCV), mean corpuscular hemoglobin (MCH) mean corpuscular hemoglobin concentration (MCHC) were seen among the experimental groups (Table 3). List separately as Table 3, Table 4, Table 5 However, the results show that there was a significant decrease in the red blood cell (RBC) count and hemoglobin level (Hb) in the control-S180 group compared to the healthy mice. Trachylobane-360 (25 mg/kg) treatment did not alter these parameters compared to baseline values in healthy mice, with values being significantly higher than those obtained for the control-S180 group (Table 4). Moreover, the animals transplanted with sarcoma 180 tumor showed a significant increase in total numbers of circulating peripheral leukocytes compared to healthy animals. There was an increase in the percentage of neutrophils and decreased percentage of lymphocytes in the peripheral blood of animals transplanted with sarcoma 180, compared to the healthy group treated only with 5% Tween-80 (Table 5). 5-FU treatment led to a decrease in the number of total circulating leukocytes, as well as the percentage of neutrophils, when compared to the tumor-being control group. Additionally, animals treated with 5-FU showed an increase in the percentage of lymphocytes compared to the control-S180 group. Trachylobane-360 treatment induced no significant change in the levels of leukocytes, or the percentages of lymphocytes and neutrophils, when compared to control-S180 group.

**Table 3:** Effects of trachylobane-360 isolated from *Xylopia langsdorffiana* on the hematological parameters determined in peripheral blood. Mice were inoculated with sarcoma 180 cells (25 × 10^6^ cells/mL, s.c.). The animals were treated for seven consecutive days, starting one day after tumor implantation.

Drug	Dose (mg/kg)	MCV ( *f*m^3^)	MCH (pg)	MCHC (g/dL)
Healthy mice				
(5% Tween-80)	-	43.40 ± 0.93	14.66 ± 0.34	33.70 ± 0.29
Control-S180				
(5% Tween-80)	-	44.50 ± 1.52	14.60 ± 0.34	32.95 ± 0.53
5-FU	25	43.67 ± 0.49	15.08 ± 0.23	34.45 ± 0.68
Trachylobane-360	12.5	42.83 ± 0.79	14.80 ± 0.30	34.63 ± 0.33
Trachylobane-360	25	43.33 ± 0.56	14.92 ± 0.13	34.60 ± 0.28

Data are presented as means ± SEM of six animals.

Patients with neoplastic diseases have a high frequency of abnormal blood cells. A decrease in hemoglobin level and red blood cell count is a common complication seen in cancer patients. The anemia present in cancer patients may have several causes, among them immune reactions, such as the production of inflammatory cytokines in response to the presence of tumor cells (known as chronic anemia of cancer) [32]. The results show that implantation of the tumor alone was capable of significantly lowering the number of red blood cells and hemoglobin level. In contrast, animals treated with 25 mg/kg trachylobane-360 showed no significant changes in blood cells numbers or hemoglobin level in relation to the healthy control group (Table 4). This means that the strong influence of the tumor to induce reduction on these parameters through different mechanisms, was reversed by treatment with trachylobane-360 (25 mg/kg), showing that even in the presence of tumor, the diterpene does not affect these parameters and therefore does not cause toxicity to blood cells and hematopoiesis.

**Table 4:** Effects of trachylobane-360 isolated from *Xylopia langsdorffiana* on the hematological parameters on peripheral blood. Mice were inoculated with sarcoma 180 cells (25 × 10^6^ cells/mL, s.c.). The animals were treated for seven consecutive days, starting one day after tumor implantation.

Drug	Dose (mg/kg)	RBC (10^6^ mm^−3^)	Hemoglobin (g/dL)	Hematocrit (%)
Healthy mice				
(5% Tween-80)	-	9.87 ± 0.25	14.46 ± 0.27	42.94 ± 0.62
Control-S180				
(5% Tween-80)	-	7.89 ± 0.36 ^c^	11.45 ± 0.73 ^c^	34.80 ± 2.35
5-FU	25	8.60 ± 0.18 ^c^	12.95 ± 0.10	37.73 ± 0.93
Trachylobane-360	12.5	8.79 ± 0.18 ^c^	13.03 ± 0.36	37.63 ± 1.10
Trachylobane-360	25	9.67 ± 0.15 ^a,b^	14.28 ± 0.18 ^a^	40.55 ± 0.57

Data are presented as mean ± SEM of six animals. ^a^ *p* < 0.05 compared to control-S180, by ANOVA followed by Tukey’s test; ^b^ *p* < 0.05, compared to mice transplanted with S180 tumor and treated with 5-FU, by ANOVA followed by Tukey’s test; ^c^ *p* < 0.05, compared to healthy mice, by ANOVA followed by Tukey’s test.

**Table 5:** Effects of trachylobane-360 isolated from *Xylopia langsdorffiana* on the hematological parameters on peripheral blood. Mice were inoculated with sarcoma 180 (25 × 10^6^ cells/mL, s.c.). The animals were treated for seven consecutive days, starting one day after tumor implantation.

Drug	Dose(mg/kg)	Total leukocytes (10^3^ mm^−3^)	Differential count of leukocytes (%)			
			Lymphocytes	Neutrophils	Monocytes	Eosinophils
Healthy mice						
(5% Tween-80)	-	7.08 ± 0.51	77.80 ± 3.73	18.40 ± 3.25	3.40 ± 0.51	0.40 ± 0.24
Control-S180						
(5% Tween-80)	-	13.34 ± 2.58 ^c^	44.00 ± 2.54 ^c^	50.50 ± 3.16 ^c^	4.33 ± 0.56	0.67 ± 0.33
5-FU	25	3.95 ± 0.51 ^a^	77.17 ± 2.18 ^a^	18.50 ± 2.32^a^	3.50 ± 0.62	0.50 ± 0.34
Trachylobane-360	12.5	11.15 ± 1.28 ^b^	51.83 ± 4.66 ^b,c^	41.17 ± 5.25 ^b,c^	6.00 ± 1.57	1.00 ± 0.45
Trachylobane-360	25	11.42 ± 0.92 ^b^	53.17 ± 5.08 ^b,c^	38.67 ± 4.56 ^b,c^	6.83 ± 1.11	0.50 ± 0.22

Data are presented as mean ± SEM of six animals. ^a^ *p* < 0.05 compared to control-S180 by ANOVA followed by Tukey; ^b^ *p* < 0.05 compared to mice transplanted with tumor S180 and treated with 5-FU, by ANOVA followed by Tukey’s test; ^c^ *p* < 0.05 compared to healthy mice, by ANOVA followed by Tukey’s test.

Tumor inoculation itself causes a leukemoid reaction in experimental animals, characterized by granulocytosis and splenomegaly [33,34,35]. The present data corroborated these findings, since animals inoculated with sarcoma 180 showed an increase in spleen weight accompanied by up-regulation of granulopoiesis. According to Okawa *et al.* [34], there is an augmentation of host resistance to fungal infection in tumor-bearing mice, and the suppression of the immune response observed in tumor-bearing animals and patients is generally caused by cancer chemotherapy, as observed in the present study when transplanted mice were treated with 5-FU. However, all hematological parameters after treatment with trachylobane-360 remained unchanged compared to the control-S180 group. These findings suggest that, unlike many chemotherapeutic agents, this diterpene does not affect hematopoietic cells, which represents an important side effect of cancer treatment.

###### 2.2.2.1.4. Histopathology Changes

**Figure 6:**
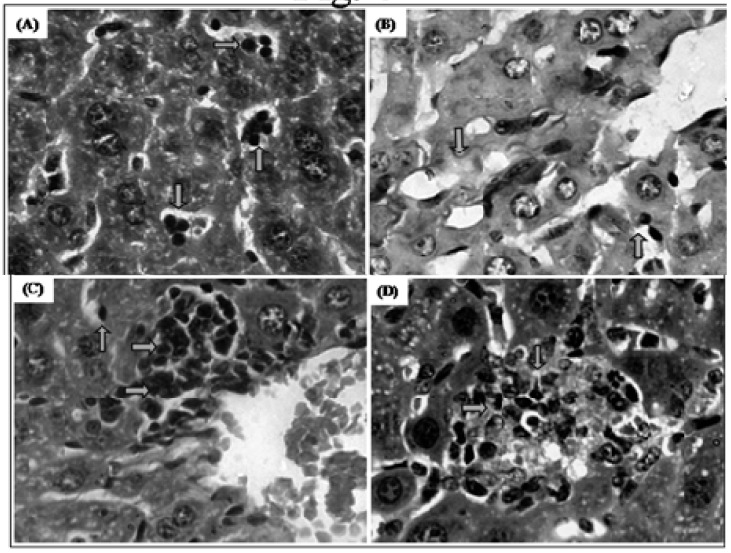
Effect of trachylobane-360 on the liver of mice transplanted with sarcoma 180 tumor. Photomicrographs show the histopathology of the livers from control-S180 (panel **A**), 25 mg/kg 5-FU-treated (panel **B**), 12.5 mg/kg trachylobane-360-treated (panel **C**), and 25 mg/kg Trachylobane-360-treated (panel **D**) analyzed by light microscopy. Histological sections stained with hematoxylin-eosin (**A**, **C** and **D**) or picrosirius red (**B**) (×400).

Histopathological analyses of livers removed from trachylobane-360-treated animals, at a dose 12.5 mg/kg, showed rare atypical regenerative hepatocellular, sinusoidal mixed inflammation, mainly in zone 3, Kupffer cell hyperplasia, inflammatory infiltrate in portal spaces (Figure 6C), parenchymal necrosis, however, characterized as mild and focal perivenulit (zone 3). In addition, at a dose 25 mg/kg, the animal livers showed multifocal parenchymal necrosis (Figure 6D). The use of the chemotherapeutic agent 5-FU at a dose of 25 mg/kg, caused multifocal parenchymal necrosis in zone 3 with production of thin collagen fibers (fibrosis), as evidenced by the special stain picrosirius red (Figure 6B). However, with the exception of parenchymal necrosis all other modifications were also observed in the control-S180 group (Figure 6A), suggesting that these effects were not related to the drug treatment alone, since the livers examined in all experimental groups showed normal lobular architecture and isomorphic hepatocytes.

**Figure 7:**
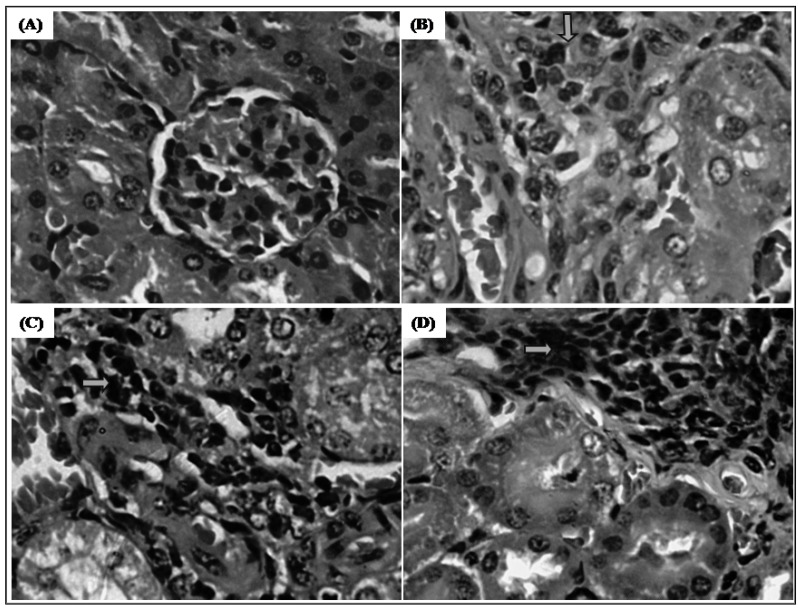
Effect of trachylobane-360 on kidneys of mice transplanted with sarcoma 180 tumor. Photomicrographs show the histopathology of the kidneys from control-S180 (panel **A**), 25 mg/kg 5-FU-treated (panel **B**), 12.5 mg/kg trachylobane-360-treated (panel **C**), and 25 mg/kg trachylobane-360-treated (panel **D**) analyzed by light microscopy. Histological sections stained with hematoxylin-eosin (×400).

Histopathological analyses of kidneys from 5-FU- and trachylobane-360-treated animals produced only chronic interstitial nephritis with lymphocytic foci. However, the structure of the glomeruli was essentially preserved (Figure 7).

The histopathological examination of the livers of control-S180 animals showed the presence of some liver reaction which may be a local consequence of a systemic effect (tumor), or may be the result of drug administration. Animals treated with 5-FU suffered greater liver damage compared to those treated with either dose of trachylobane-360. However, in both trachylobane-360 groups, no changes occurred long enough to cause the release of liver enzymes into the plasma.

In this study, histopathological analysis of the kidneys of treated animals showed the presence of similar changes in all groups, but these were not extensive enough, since the glomeruli, Bowman's capsule, mesangial cells, proximal convoluted tubules, loop of Henle and distal convoluted tubules were preserved without detrimental histological features, which corroborates the results for the biochemical parameters of renal function, showing no change in urea or creatinine levels.

## 3. Experimental

### 3.1. Drug

Trachylobane-360, *ent*-7α-acetoxytrachyloban-18-oic acid, was purified from *Xylopia langsdorffiana* stems as described by Tavares *et al.* [6] (Figure 1). Dried stems of *X. langsdorffiana* (4 kg) were exhaustively extracted with 95% EtOH (3 × 5 L) for 72 hours at room temperature The solvent was evaporated to yield a dark syrup (60 g), which was successively partitioned with hexane, chloroform and ethyl acetate to yield 20, 16, and 12 g of crude residue, respectively. The hexane fraction was subjected to column chromatographic separation, using hexane and hexane with increasing amounts of ethyl acetate as eluents, and the eluate was monitored by TLC. Altogether, 95 fractions of 100 mL each were collected and pooled into 12 fractions (F-1-F-12). Fraction F-1 was recrystallized from methanol, yielding trachylobane-360 (300 mg). Trachylobane -360, obtained as a pure compound, was characterized particularly by 1D and 2D NMR, X-ray crystallography and m.p. of 230–233 °C. 5-FU was purchased from Sigma Aldrich Co. (St. Louis, MO, USA).

### 3.2. Animals and Tumor Cells

Sarcoma 180 tumor cells were maintained in the peritoneal cavity of Swiss mice in the Laboratory of Toxicological Assays at the Federal University of Paraiba, Brazil. For the *in vitro* cytotoxicity assays, the cell line was cultured in RPMI-1640 supplemented with 10% fetal bovine serum, 2 mM glutamine, 100 U/mL penicillin, 100 μg/mL streptomycin and 2 mM HEPES, at 37 °C with 5% CO_2_.

A total number of 30 Swiss mice (female, 28–32 g) obtained from the Bioterum Prof. Thomas George of the Federal University of Paraiba, Brazil, were used. Animals were housed in cages with free access to food and water. All animals were kept under a 12 h:12 h light-dark cycle (lights on at 6:00 a.m.). Animals were treated according to the ethical principles of animal experimentation of COBEA (Colégio Brasileiro de Experimentação Animal), Brazil. The Animal Studies Committee of Universidade Federal da Paraíba approved the experimental protocol.

### 3.3. Hemolysis Assay

The hemolytic activity of trachylobane-360 was tested using mouse erythrocytes according to Kang *et al.* [36], with some modifications. Briefly, fresh blood samples were collected with a heparinized capillary to prevent blood coagulation. To obtain a pure suspension of erythrocytes, 1.5 mL of whole blood was then made up to 10 mL in phosphate buffered saline (PBS, pH 7.4), and centrifuged at 3,000 rpm for 5 min. The supernatant and buffy coat were then removed by gentle aspiration, and the above process was repeated two more times. Erythrocytes were finally resuspended in PBS to make 1% solution for the hemolysis assay. Various concentrations of trachylobane-360 (0–555 µM), dissolved in DMSO (5%), were added to the suspension of red blood cells obtained from mice. The tubes with the trachylobane-erythrocyte mixtures were incubated on a mixer for 60 min and then centrifuged at 3,000 rpm for 5 min. The absorbance of the supernatants was determined at 540 nm using a UV-Vis Spectrophotometer (UV-1650PC Shimadzu^®^) to measure the extent of red blood cell lysis and determine the concentration that produces 50% hemolysis (HC_50_). Positive control (100% hemolysis) and negative control (0% hemolysis) were also determined by incubating erythrocytes with 1% Triton X-100 in PBS and PBS alone, respectively.

### 3.4. Antitumor Activity of Trachylobane-360

#### 3.4.1. Determination of the Effect of Trachylobane-360 on Tumor Cells in Culture

Ten-day-old Sarcoma 180 ascites cells were added to PBS and subsequently centrifuged (1,000 rpm for 10 min). The supernatant was discarded and cells were resuspended in RPMI-1640 supplemented with 25 mM HEPES, 2 mM L-glutamine, 100 U/mL penicillin, 100 µg/mL streptomycin and 10% fetal bovine serum. The cells were then seeded (2 × 10^5^ cells/well suspended in 100 μL of medium) in 96-well plates and incubated with different concentrations of trachylobane-360 for 24 h (37 °C and 5% CO_2_). The compound was first dissolved in dimethylsulfoxide (DMSO) and then in supplemented medium. The final concentration of DMSO in the test medium and controls was 0.1%. Two cytotoxicity assays were used: trypan blue (TB) exclusion test and MTT reduction assay. The TB exclusion test determines the number of viable cells with intact plasma membrane, capable of excluding the TB dye. The TB exclusion assay was performed as described by Renzi *et al.* [37]. After 24 h incubation with trachylobane-360, TB solution (0.4% in PBS) and cell suspension were mixed in equal volumes (0.01 mL) and the number of cells was estimated using a hemocytometer. Cells stained blue were scored as dead. The MTT reduction assay determined the number of living cells able to reduce the yellow dye 3-(4,5-dimethyl-2-thiazolyl)-2,5-diphenyl-2*H*-tetrazolium bromide (MTT) to a purple formazan product [38]. Briefly, 0.01 mL of serum-free medium containing MTT (5 mg/mL) was added to each well. After incubation for 4 h, the blue formazan product obtained was dissolved in 0.05 mL of sodium dodecylsulfate (10%) in 0.1 N HCl with stirring for 10 min on a microplate shaker, after which the absorbance at 570 nm was read using a multiplate reader (TP Reader–ThermoPlate^®^, Shenzhen, China). The concentration that caused 50% cell growth inhibition (IC_50_) was determined in the two assays.

#### 3.4.2. Determination of the Effect of Trachylobane-360 on Tumor Growth *in vivo*

Ten-day-old sarcoma 180 ascites cells (25 × 10^6^ cells/ml) were implanted subcutaneously into the left subaxillary region of the mice [31]. One day after inoculation, trachylobane-360 (12.5 or 25 mg/kg) was dissolved in 5% Tween-80 and administered intraperitoneally for 7 days in mice transplanted with sarcoma 180 tumor. 5-FU (25 mg/kg) was used as a positive control. The control-S180 group (mice bearing sarcoma 180) and healthy control (healthy mice) were inoculated with 5% Tween-80 in 0.9% NaCl. On day 8, peripheral blood samples from control and treated mice were collected from the retro-orbital plexus under light sodium thiopental anesthesia, and animals were then sacrificed by cervical dislocation. The tumors were excised and weighed, and then fixed in 10% formaldehyde and submitted to histopathological analysis. The rate of tumor growth inhibition (%) was calculated by the following formula: 


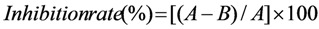
(1)

where A is the tumor weight average of the negative control, and B is that of the treated group.

##### 3.4.2.1. Toxicological Analyses

###### 3.4.2.1.1. Determination of the Effect of Trachylobane-360 on Body and Organ Weight

Body weights were measured at the beginning and end of the treatment, and the animals were observed for signs of abnormalities throughout the study. The liver, spleen, thymus, and kidneys were excised and weighed.

###### 3.4.2.1.2. Determination of the Effect of Trachylobane-360 on Biochemical Parameters

For biochemical analysis, the blood samples from control and treated mice were centrifuged at 3,500 rpm for 10 min, and the levels of urea, creatinine, alanine aminotransferase (ALT) and aspartate aminotransferase (AST) were determined. These levels were determined with the COBAS Mira Plus analyzer (Roche Diagnostic Systems, Basel, Switzerland) using standardized diagnostic kits (LABTEST®).

###### 3.4.2.1.3. Determination of the Effect of Trachylobane-360 on Hematological Parameters

For the hematological analysis, an aliquot of blood was collected from each animal with a heparinized capillary, and various hematological parameters (hemoglobin (Hb) level, red blood cell (RBC) count, hematocrit (Hct) and red cell indices as mean corpuscular volume (MCV), mean corpuscular hemoglobin (MCH) and mean corpuscular hemoglobin concentration (MCHC), and total and differential leukocyte count) were carried out by standard procedures using an automatic hematological cell analyzer (Animal Blood Counter–ABC Vet, Montpellier, France) and light microscopy.

###### 3.4.2.1.4. Histopathological Analyses

After being weighed and fixed in 10% formaldehyde, tumors, livers and kidneys were grossly examined for size or color changes and hemorrhage. Portions of the tumors, livers, and kidneys were cut into small pieces, followed by staining of the histological sections with hematoxylin-eosin. Moreover, for detection of hepatic fibrosis, the livers were also stained with picrosirius red. Histological analysis was performed by light microscopy to determine the presence and extent of liver or kidney lesions attributed to drugs.

### 3.5. Statistical Analysis

Data are presented as means ± S.E.M. The HC_50_ and IC_50_ values and their 95% confidence intervals (CI 95%) were obtained by nonlinear regression. The differences between experimental groups were compared by analysis of variance (ANOVA) followed by Tukey’s test (*p* < 0.05) using the GRAPHPAD program (Intuitive Software for Science, San Diego, CA, USA).

## 4. Conclusions

In conclusion, the data presented here reinforce the anticancer potential of natural products, since trachylobane-360 caused *in vitro* and *in vivo* growth inhibition of tumor cells, without major changes in biochemical, hematological and histopathological parameters. 
